# Supplementary material for: Left atrial emptying fraction determined during atrial fibrillation predicts maintenance of sinus rhythm after direct current cardioversion in patients with persistent atrial fibrillation
Source: PLoS One. 2020 Aug 21;15(8):e0238002. doi: 10.1371/journal.pone.0238002 (PMC7446797; doi:10.1371/journal.pone.0238002)
Supplement: Attachment — Submitted filename: Reviewer II.docx [file pone.0238002.s002.docx]

Dear Reviewer,

Thank you very much for a thorough review of our manuscript and valuable comments. We would also like to apologize for minor editorial errors. We hope that our amendments will meet your acceptance.

We responded to the comments as follows:

1. Patients with high ventricular response to atrial fibrillation were counted in the exclusion criteria? Under this circumstance, the assessment of left atrial function is challenging. A cut-off of mean ventricular rate was taken into account?

Thank you for your comment. Due to the fact that all patients included in the study were patients admitted to elective DCCV, they had optimally controlled ventricular rate. Due to the significant value of information about the ventricular rate, we added "ventricular rate greater than 120 beats per minute" to the exclusion criteria because among the patients in our study the upper limit of the average ventricular rate during AF before DCCV was 120 beats per minute.

In the study population chapter, the sentence has been changed.

“Exclusion criteria were as follows: age under 18 years, lack of consent for study participation, lack of consent for DCCV, poor quality of echocardiography visualization, ventricular rate greater than 120 beats per minute, moderate or severe valve regurgitation or stenosis…”.

2. In the Results section, the number of patients with sinus rhythm maintenance/recurrence of atrial fibrillation is not clear with incongruity between the text and the Tables 1 and 2.

The sentences have been corrected. We apologize for this mistake.

“Of the 146 patients enrolled in the study, 61 (41,8%) patients maintained SR after 12 months of follow-up”.

3. In the paragraph “Atrial enlargement” LAESVI is mentioned many times, instead of left atrial end-diastolic volume index (LAEDVI).

The sentences have been corrected. We apologize for the mistake.

“In the studied population, the mean LAVI was 47.8±12.4 ml/m², the mean LAEDVI was 36.6±12.3 ml/m², and the mean LAAP diameter was 44±4.5 mm. Patients with a smaller LA cavity were more likely to maintain SR. The group with SR maintenance and the group with AF recurrence had statistically significant differences in LAVI (44.3±11.5 vs. 50.3±12.5ml/m²; p=0.004) and LAEDVI (30.5±11 vs. 37.6±12.5 ml/m²; p<0.001), but there was no difference in LAAP diameter”.

4. In the same paragraph, there are some mistakes in the statistical connections of the sentence “The univariate logistic regression analysis revealed that elevated values of LAVI and LAEDVI decreased the odds of maintaining SR, with an odds ratio (OR) of 0.958 (95%CI 0.929-0.987; p=0.006) for the patients that maintained SR and 0.946 (95%CI 0.916-0.978; p=0.001) for those with AF recurrence”.

The sentences have been corrected. We apologize for the mistake.

“The univariate logistic regression analysis revealed that elevated values of LAVI and LAEDVI decreased the odds of maintaining SR, with an odds ratio (OR) of 0.958 (95%CI 0.929-0.987; p=0.006) and 0.946 (95%CI 0.916-0.978; p=0.001) respectively”.

5. In the Abstract, the sentence “Analysis with a model including clinical and echocardiographic variables, only LAEF …” is clearer writing “Analyzing a model including clinical and echocardiographic variables, only LAEF …”.

The sentences have been corrected.

6. Make sure to report the same numerical data in the text and in Tables; there are a few discrepancies between the data typed in the text and in the Tables (for example: page 9: 37.6±12.5 ml/m2 in the text vs 37.6±12.3 ml/m2 in Table 2; page 10: 95%CI 0.735-0.938 in the text vs 95%CI 0.753-0.938 in Table 3).

The sentences have been corrected.

7. At page 9 there is a typo: 95%CI 0.8859-1.012, correct form: 0.859.

The sentence has been corrected.

8. At page 9 the sentence “In the univariate analysis, the OR for LAEF for the SR maintenance group was…” is clearer writing “In the univariate analysis, the OR of LAEF for SR maintenance was …”.

The sentence has been corrected.

9. In the Figure legends at page 12 probably there is a typo: “p values”.

The sentences have been corrected.

10. In the last sentence of the Conclusions, the correct form is “ventricular rate control”.

The sentence has been corrected.

Yours faithfully,

Authors
